# Supplementary material for: Spatial patterns and predictors of missing key contents of care during prenatal visits in Ethiopia: Spatial and multilevel analyses
Source: PLoS One. 2024 Nov 18;19(11):e0313893. doi: 10.1371/journal.pone.0313893 (PMC11573206; doi:10.1371/journal.pone.0313893)
Supplement: Attachment — Submitted filename: comment1.pdf [file pone.0313893.s003.pdf]

1. Your source data is outdated, from 2016. How important is it for policymakers to consider the changes that have occurred since then? The Ministry of Health has implemented several programs, such as increasing accessibility to laboratories and prioritizing services for antenatal care (ANC) mothers.
2. The study did not reveal any significant new variables that were previously unknown. What novel information has your study provided?

3. What suggestions do you have for other researchers?

4. Is it feasible for policymakers to enhance maternal basic education? If so, how can they achieve this?

5. Your final result is influenced by the way you define and measure your variables, so it is important to conduct a reanalysis after making any corrections to your operational definitions.

   “If a woman missed all or at least one of the seven items of care, she was considered to have missed the contents of care, and the woman was only considered to have full contents of care if she 146 received all seven items of care.
6. Have you taken into account other factors such as waiting time, healthcare worker ethics, and so on?
7. What is the clear gap identified in the previous study?
8. Further elaboration is required in the discussion section, as well as a more comprehensive exploration of the limitations
