# Supplementary material for: Spatial patterns and predictors of missing key contents of care during prenatal visits in Ethiopia: Spatial and multilevel analyses
Source: PLoS One. 2024 Nov 18;19(11):e0313893. doi: 10.1371/journal.pone.0313893 (PMC11573206; doi:10.1371/journal.pone.0313893)
Supplement: Attachment — Submitted filename: Response to reviewers.docx [file pone.0313893.s002.docx]

**A point-by-point response to editor and reviewers**

**Authors’ Response to Academic Editor**

**Dear: Agmasie Damtew Walle, Academic Editor, Plos One**

We thank you for a thorough reading and constructive comments and suggestions on our manuscript and for the opportunity to revise and resubmit. We are pleased to submit the revised version of the manuscript titled “**Spatial patterns and predictors of missing key contents of care during prenatal visits in Ethiopia: Spatial and multilevel analyses**” for your consideration in the special collection of **Plos One**. The comments of the editors and the reviewers were highly insightful and enabled us to greatly improve the quality of our manuscript. In this revised manuscript we made substantial changes to address your concerns in a point-by-point response. We appreciate your time and look forward to your response and we are very keen to incorporate further comments, if any, for the betterment of the final manuscript.

On the following pages, you will find our responses to the comments and suggestions raised by the esteemed editor and reviewer.

Sincerely,

Aklilu Habte(MPH)(corresponding author)

[**aklilihabte57@gmail.com**](mailto:aklilihabte57@gmail.com)

**Response to Journal requirements**

**1.** Please ensure that your manuscript meets PLOS ONE's style requirements, including those for file naming.

**Response:** we already prepared the manuscript as per the journal requirement and again we rechecked the compliance towards it during the submission of our revised manuscript.

**2.** We note that Figures 2, 4 and 5 in your submission contain map images which may be copyrighted. All PLOS content is published under the Creative Commons Attribution License (CC BY 4.0), which means that the manuscript, images, and Supporting Information files will be freely available online, and any third party is permitted to access, download, copy, distribute, and use these materials in any way, even commercially, with proper attribution. For these reasons, we cannot publish previously copyrighted maps or satellite images created using proprietary data, such as Google software (Google Maps, Street View, and Earth).

We require you to either (1) present written permission from the copyright holder to publish these figures specifically under the CC BY 4.0 license, or (2) remove the figures from your submission:

You may seek permission from the original copyright holder of Figures 2-9 to publish the content specifically under the CC BY 4.0 license.

**Response:** We appreciate your concern to assure the ethical issues. However, all the aforementioned figures(2.4, and 5) in our manuscript are not copyrighted rather they are the result of spatial analysis that we have run in ArcGIS and SaTScan software. The GPS and DHS data that contain Shapefile and other relevant variables were obtained from the DHS office by explaining the objective of the study through online requests. Then, in order to get those figures, we import the relevant data extracted from the 2016 Ethiopian Demographic Health Survey reports and the shapefile of Ethiopia obtained from the 2016 Ethiopian Central Statistical Agency (CSA).To indicate this, we already cited the source of the shapefile alongside each figure. The shape file that we used to construct the figures can be accessed by one of the following links:

1. <https://data.humdata.org/dataset/cb58fa1f-687d-4cac-81a7-655ab1efb2d0> .
2. <https://gadm.org/download_country.html>

Therefore, the maps presented in our study are not copyrighted rather they were the outputs of our spatial analysis results which are the result of those Shapefiles and projected CVS files in ArcGIS. This is the actual procedure that we employed in our present and earlier studies, as well as other Ethiopian researchers. Again we assure you that the figures presented in our study are not copyrighted but rather our spatial analysis results.

**3.** Please include captions for your Supporting Information files at the end of your manuscript, and update any in-text citations to match accordingly. Please see our Supporting Information guidelines for more information: <http://journals.plos.org/plosone/s/supporting-information>

**Response:** Thank you for the suggestion. However, we do not have any supporting information to cite; all of the results were brought up in the manuscript.

**Response to Additional Editor Comments:**

**Comment 1:** why use Ordinary Kriging interpolation for prediction of missing ANC content

**Response:** Thank you for your insightful inquiry. The possible justifications behind using an ordinary Kriging interpolation were:

1. It takes into account the spatial dependence or autocorrelation in the data. In the context of ANC content, it recognizes that nearby locations are likely to have similar values. This is important for accurately predicting missing values based on the values observed at surrounding locations.
2. It minimizes the prediction variance by giving more weight to nearby sample points with similar values. This helps in obtaining more reliable predictions, especially in areas where the data is sparse.
3. It provides an optimal linear unbiased estimate of the variable being interpolated by combining the information from neighboring points to estimate the value at a target location while minimizing the bias in the estimation.
4. Furthermore, because ordinary Kriging is based on geostatistical models that account for both spatial trend and spatial variability in data, it provides a more accurate depiction of the underlying spatial patterns in ANC content.

We have added all the possible justifications and highlighted them in the ‘spatial analysis’ section of the "Revised Manuscript with Track Changes" Page 7, Lines 181-187

**Comment 2:** what is the reason to select The Bernoulli-based approach was used to find statistically significant spatial clusters with a high number of women who missed the content of care**.**

**Response:** Thank you for asking this are the reasons for using the model:

1. The Bernoulli model allows for flexibility in defining the event of interest. In this case, it can be easily adapted to identify spatial clusters of women who missed the content of antenatal care.
2. Furthermore, the results of the Bernoulli-based spatial scan analysis tend to be flexible and easy to interpret, such as revealing clusters with high or lower risk factors, making it easier for policymakers and public health experts to understand and focus interventions.

The details were added and highlighted in the ‘Spatial Scan Statistical Analysis’ section of the "Revised Manuscript with Track Changes" Page 7, Line 195-201

**Comment 3:** why not conduct the cluster outlier instead of hotspot analysis

**Response:** Thank you for your insightful inquiry. Hotspot analysis is known to identify areas with statistically significant clusters of high or low values of missing key ANC contents., and it helps in understanding spatial patterns and trends within the data. In addition, Hotspot analysis often involves techniques like Getis-Ord Gi* statistic or Moran's I, which assess the spatial autocorrelation and significance of clustered patterns. On the other hand, cluster outlier analysis focuses on identifying specific locations that deviate significantly from the spatial pattern observed in the dataset.

Our reasons behind prefering Hotspot Analysis are:

- Hotspot analysis is better suited for identifying overall patterns and trends in the data by allowing us to understand where clusters of high or low values are located, which provides a clearer picture.
- When the goal is to inform policy or interventions, hotspot analysis is more appropriate. It helps in identifying areas where targeted efforts or resources may be needed.
- Hotspot analysis provides both global and local insights. Global measures identify overall spatial patterns, while local measures pinpoint specific clusters.

Overall, a hot spot is more preferable one and almost all of the recent studies in Ethiopia and other African countries performed the spatial one.

**Comment 4:** Please incorporate in the introduction section why you want to study this topic in detail

**Response:** thank you for your insightful suggestion to incorporate the rationales of the study. Accordingly, we have added the possible reasons behind conducting the current study and highlighted them in the ‘introduction’ section of the "Revised Manuscript with Track Changes" Page 4, Line 102-106

**Comment 5:** Include the inclusion and exclusion criteria

**Response:** we appreciate your inquiry. We have incorporated all the eligibility criteria in the ‘Data source and population’ section of the "Revised Manuscript with Track Changes" Page 4, Lines 119-120.

**Comment 6:** please mention what are the gaps that you identified from the previous study

**Response:** thank you for your insightful suggestion. The main gap that we identified from previous studies was they primarily focused on the timing and number of visits, and the level of adequacy of services received during visits with their spatial variation has not been thoroughly assessed. Thus, we have mentioned and highlighted this as the main gap and rationale for conducting the current study, in the ‘introduction’ section of the "Revised Manuscript with Track Changes" Page 4, Line 102-106

**Comment 7:** Please revise the discussion section clearly

**Response:** Thank you for your suggestion. Accordingly, we thoroughly look into the discussion section again and we have made some amendments. The statements with slight modifications were highlighted throughout the ‘Discussion’ section of the "Revised Manuscript with Track Changes"

Thank you for your constructive comments and suggestions, which we got as valuable input in the improvement of the manuscript. We received all of them as a valuable contribution to our ongoing work. In the following section, we tried to respond to all the possible comments and suggestions from reviewer #1

_____________________________END________________________________________

THANK YOU!!!

**Authors’ Response to Reviewer**#**1**

Dear Reviewer 1, thank you very much for taking the time to review our work and for your positive feedback. We received your thoughtful, and generous review, along with helpful feedback and suggestions, as a valuable contribution to our ongoing work. We have tried to address all the possible comments and suggestions raised by you in the following session.

**General comments**:

**Comment 1:** better to include line numbers in the manuscript for smooth communication.

**Response:** A sincere apology for not incorporating line numbers for the manuscript and making the review process clattering. As per your suggestion, we have added the line number to the revised version of the manuscript and we gave the responses by mentioning the line and page numbers.

Comment 2: In the variable subsection, the Authors mentioned "Based on the responses, a composite index was created with minimum and maximum values of 0 and 7, respectively. Finally, the count variable was dichotomized as “1” if any of the seven key contents of care were missed and “0” if she received all the contents of care". Does it scientifically sound to to say 1 if a woman missed only one component? Does it mean missing one component and all the seven key contents are the same? I see a significant information loss. I think it should be written in a persuasive way to avoid this confusion.

**Response:** Thank you for your thoughtful and thorough evaluation. We meant that a woman who missed at least one of the seven items of care was regarded to have missed the contents of care. In contrast, the woman is deemed to have full contents of care only if she received all seven contents of care. The reason behind using such a clear-cut classification is to show the level of the quality of ANC, and that might be the reason the prevalence became high(88.2%).

We have re-write the statement and highlighted it in the ‘Variables of the study’ section of the "Revised Manuscript with Track Changes" Page 5, Line 141-146

Thank you for your constructive comments and suggestions, which we got as valuable input in the improvement of our manuscript. We received all of them as a valuable contribution to our ongoing work.

**_________________________________________________________________________________________________________END_______________________________________**

**THANK YOU!!!**
