# Supplementary material for: Spatial patterns and predictors of missing key contents of care during prenatal visits in Ethiopia: Spatial and multilevel analyses
Source: PLoS One. 2024 Nov 18;19(11):e0313893. doi: 10.1371/journal.pone.0313893 (PMC11573206; doi:10.1371/journal.pone.0313893)
Supplement: Attachment — Submitted filename: Response to reviewer.docx [file pone.0313893.s004.docx]

**A point-by-point response to editor and reviewer**

**Authors’ Response to Academic Editor**

**Dear: Avanti Dey, PhD, Staff Editor, Plos One**

We thank you for a thorough reading and constructive comments and suggestions on our manuscript and for the opportunity to revise and resubmit. We are pleased to submit the revised version of the manuscript titled “**Spatial patterns and predictors of missing key contents of care during prenatal visits in Ethiopia: Spatial and multilevel analyses**” for your consideration in the special collection of **Plos One**. The comments of the editors and the reviewers were highly insightful and enabled us to greatly improve the quality of our manuscript. In this revised manuscript we made substantial changes to address your concerns in a point-by-point response. We appreciate your time and look forward to your response and we are very keen to incorporate further comments, if any, for the betterment of the final manuscript.

On the following pages, you will find our responses to the comments and suggestions raised by the esteemed reviewer.

Sincerely,

Aklilu Habte(MPH)(corresponding author)

[**aklilihabte57@gmail.com**](mailto:aklilihabte57@gmail.com)

**Authors’ Response to Reviewer**#**1**

Dear Reviewer 1, thank you very much for taking the time to review our work and for your positive feedback. We received your thoughtful, and generous review, along with helpful feedback and suggestions, as a valuable contribution to our ongoing work. We have tried to address all the possible comments and suggestions raised by you in the following session.

**Comment 1:** Your source data is outdated, from 2016. How important is it for policymakers to consider the changes that have occurred since then? The Ministry of Health has implemented several programs, such as increasing accessibility to laboratories and prioritizing services for

antenatal care (ANC) mothers.

**Response:** we appreciate your insightful inquiry. We used the 2016 EDHS report since there was no other standard DHS report in Ethiopia, and it was the most recent and relied on a large number of respondents, and the reports from the current study may serve as a basis for evaluating future DHS reports. Initially, we planned to perform the study using the 2019 mini-demographic report, but it lacks the majority of the variables included in the current study, and we were unable to obtain the majority of the contents of care provided during prenatal care. After evaluating both of those challenges, we decided to conduct our analysis on the 2016 report. To obtain the most recent data, we may undertake the study during the upcoming series of standard DHS in 2025/26. Furthermore, the majority of studies that relied on DHS report in Ethiopia used 2016 one.

**Comment 2:** The study did not reveal any significant new variables that were previously unknown. What novel information has your study provided?

**Response:** The following were some of the novel perspectives in the current study.

1. There was a dearth of studies that showed geographical variation in missing adequate contents of care during prenatal visits (inadequacy of care).
2. Previous studies conducted in Ethiopia using DHS reports merely focused on timing(1^st^ Trimester booking) and adequacy of visits(4 or more ANC visits), and they didn’t give due emphasis to missing key contents of care during ANC, which is one of the quality indicators.
3. Also, we tried to incorporate all variables at the individual and community level
4. Unlike previous studies, this study focused on missing care contents by setting a higher standard; by involving women who missed even one of the necessary seven cares in the category of 'missed care', which may reveal the actual status of ANC quality in the country.

Comment 3: What suggestions do you have for other researchers?

**Response:**

**Comment 4:** Is it feasible for policymakers to enhance maternal basic education? If so, how can they achieve this?

Response: Yes, they can strengthen basic education in two ways. To begin, they may prioritise female education by increasing adolescent girls school enrollment and reducing dropout rates, which could increase the number of educated women in the population. Second, they can strengthen informal education for women at the community level through community health workers and local administrators, which has already been implemented in several regions of the country.

**Comment 5:** Your final result is influenced by the way you define and measure your variables, so it is important to conduct a reanalysis after making any corrections to your operational definitions.

“If a woman missed at least one of the seven items of care, she was considered to have

missed the contents of care(=1), and the woman was only considered to have full contents of care(=0) if she received all seven items of care.

**Response:** Thank you very much for letting us make the operational definition more clear for the readers. Accordingly, we have made slight amendments to the operational definition and highlighted it in the ‘Variables of the study’ section of the ‘Revised Manuscript with Track changes’, Lines 141-144, Page 5. As a result, our title, method, results, and discussion were based on the women who missed the contents of care. We politely suggest you to look at the manuscript.

**Comment 6:** Have you taken into account other factors such as waiting time, healthcare worker ethics, and so on?

**Response:** thank you for asking. However, the study has relied on the EDHS data and we didn’t have any opportunity to consider those variables and we might add this as the limitation of this study in the ‘Discussion’ section of the ‘Revised Manuscript with Track changes’, Lines 432-435, Page 18.

**Comment 7:** What is the clear gap identified in the previous study?

**Response:** These were some of the gaps:

1. They didn't show geographical variation in missing adequate contents of care during prenatal visits (inadequacy of care),
2. They merely focused on timing(1^st^ Trimester booking) and adequacy of visits(4 or more ANC visits), and they didn’t give due emphasis to missing key contents of care during ANC, which is one of the quality indicators, and
3. They also failed to show which content of care was missed by most women and in which region in the way that we presented in Table 4. Thus we tried to address those gaps in this study.

**Comment 8:** Further elaboration is required in the discussion section, as well as a more comprehensive exploration of the limitations.

**Response:** thank you for your suggestion. Accordingly we tried we tried to add some elaborations and possible justifications in the discussion section and we highlighted them in the ‘Revised Manuscript with Track changes’. In addition we add another limitation in the ‘Discussion’ section of the ‘Revised Manuscript with Track changes’, Lines 432-435, Page 18.

Thank you for your constructive comments and suggestions, which we got as valuable input in the improvement of our manuscript. We received all of them as a valuable contribution to our ongoing work.

**_________________________________________________________________________________________________________END_______________________________________**

**THANK YOU!!!**
